# The use of ATR-FTIR to track the degradation of single-use polystyrene cup lids during 24 months of temperate outdoor exposure

**DOI:** 10.1371/journal.pone.0330354

**Published:** 2025-08-20

**Authors:** Megan M. Trusler, Matthew S. Kent, Barry H. Lomax, Christopher H. Vane, Sarah Cook

**Affiliations:** 1 School of Biosciences, University of Nottingham Sutton Bonnington Campus, Loughborough, United Kingdom; 2 British Geological Survey, Organic Geochemistry Facility, Keyworth, Nottingham, United Kingdom; 3 School of Life Sciences, University of Warwick, Coventry, United Kingdom; Wadi International University, SYRIAN ARAB REPUBLIC

## Abstract

A set of unused virgin polystyrene coffee cup lids were distributed in the environment (Sutton Bonington, UK) for a period of 24 months to compare monthly degradation rates across four treatments with variable degrees of exposure to natural UV irradiance (full or reduced exposure) and soil (surface or buried). Analysis of monthly samples (hole-punched discs) from three lids of each treatment via FTIR-ATR indicated that the lids in each treatment displayed varying levels of degradation, ranked as follows: exposure on the ground surface, no shading > exposure on the ground surface, shading> both buried treatments. Principal component analyses (PCAs) and the carbonyl index indicated that photooxidation via sunlight exposure was the primary degradation mechanism for polystyrene under these environmentally relevant conditions. Monthly variations in spectra for each treatment (particularly surface treatments) also indicated that degradation rate was not a continuous process, with a multiple regression establishing correlation between monthly carbonyl index for the first 12 months of the experiment, and both UV irradiance and temperature (p = 0.058). This demonstrated that environmental polystyrene degradation rate was closely related to seasonal cycles in the temperate environment.

## 1. Introduction

Plastics are widely used synthetic polymers, first achieving commercial significance in the 1930s [1]. Their success as a material is attributed to their low production costs and versatile properties that make plastics ideal for mass production including low mass, water-resistance, and long-lasting, shatter-resistant durability [2,3]. However, these same properties mean that conventional oil-based plastic items are generally inert and resistant to processes of degradation, possibly taking anywhere between hundreds or thousands of years to break down [4]. Current trends in plastics consumption indicate that approximately 12 billion tonnes of plastic will have accumulated in the environment and landfill sites by 2050 [5].

As plastics slowly age in the environment, there are a number of chemical, physical, and biological reactions which result in changes to the properties of the polymer, known as degradation. Degradation ultimately results in bond scissions and other chemical transformations that produce structural irregularities within the polymer. These processes also result in reduced visual and mechanical integrity, for example cracking, discolouration, and decreases in elasticity and tensile strength [6,7]. Depending on the conditions a plastic is exposed to, there are numerous abiotic and biotic degradation pathways including photooxidative degradation, thermal degradation, mechanochemical degradation, and biodegradation. Photooxidative degradation is thought to be one of the main sources of damage to a plastic under ambient conditions [7–10]. This process begins when exposure to light in the form of ultraviolet (UV) radiation and atmospheric oxygen causes bond dissociation within the polymer chain. This then leads to a free radical chain reaction that causes either crosslinking or chain scission, and ultimately terminates when more stable oxidised products (such as carbonyls) are formed. As a result, photooxidative degradation is represented by an increased proportion of oxygen-rich functional groups and an overall reduction in the molecular weight of the polymer [7–10]. Photooxidative degradation can be quantified using Fourier-Transform Infrared (FTIR) spectroscopy by comparing the FTIR absorbance of the carbonyl peak (C = O) to a reference peak (known as the carbonyl index).

Polystyrene (PS) is a widely used polymer that accounts for 6% of the global plastic market share, although its degradation has been investigated less than other thermoplastic polymers such as polyethylene (PE) and polypropylene (PP) [11,12]. It is commonly used in the manufacture of disposable single-use items such as plastic coffee cup lids, at least 2.5 billion of which are thrown away each year in the UK [13]. PS is an addition polymer generated from the monomer styrene, and consists of a carbon backbone with an aromatic ring (phenyl group) attached to every other carbon atom [9]. In the photooxidative degradation of PS, carbonyl compounds (ketones and aldehydes) are generated after excitation from UV radiation generates a peroxy radical through the addition of oxygen. This then leads to a chain reaction that ultimately causes chain scission and the formation of carbonyl compounds [9,10]. Although these processes have previously been explored under laboratory conditions, there are few studies that have investigated the breakdown of plastics under environmentally relevant conditions and timescales [14–16].

Understanding how PS degrades in the environment is important for understanding how it is likely to both react to and interact with the environment through its lifetime, following exposure to different conditions. While there are a number of studies that have simulated degradation under accelerated conditions, there are few that have investigated degradation pathways under natural environmentally relevant natural conditions. In this study, purchase PS coffee cup lids were exposed for 24 months to different environmental conditions they were likely to experience after they have been discarded. FTIR spectroscopy was used to track chemical changes in the spectra of the PS lids over time, with the aim of using the technique to elucidate the effect of natural UV exposure in a temperate climate on the photooxidative degradation rate of the PS polymer. Given that FTIR is one of the most widely applied characterisation techniques used in the environmental assessment of microplastics in the environment, its application to degradation studies is particularly relevant. As part of this, site UV-B, rainfall, and soil temperature fluctuations throughout the 24 months were also measured and compared to the degradation rate of each treatment. This will test the hypotheses that polystyrene exposed to the natural environment will degrade over time and that those exposed to natural irradiance show more signs of degradation in FTIR spectra (decreased absorbance at native peaks, and increased absorbance at the carbonyl wavenumber) compared to those under shade and those buried under soil, as related to photooxidative mechanisms. It will then assess the hypothesis that seasonal variation in irradiance leads to variation in the degradation rate of polystyrene.

## 2. Materials and methods

### 2.1. Study design

A total of 48 PS coffee cup lids were deployed across four treatments (12 lids per treatment) to compare the degradation of PS when exposed to different environmental conditions. The commercially available lids were purchased unused with minimal environmental exposure, made of an amorphous PS, coloured white, and considered typical examples of coffee cup lids in current circulation (average diameter: 94 mm, thickness: 0.25 mm, and average mass 2.8 g). The treatments were:

1. Placed on the ground surface and fully exposed to irradiance (surface without shade treatment)
2. Buried to 10 cm without shade (buried without shade treatment)
3. Placed on the ground surface under shade (surface with shade treatment)
4. Buried to 10 cm with shaded soil (buried with shade treatment)

Twelve lids were deployed per treatment, and three of them were sampled from within this study. In future experiment years of this ongoing study, it is anticipated that all twelve lids will need to be successively sampled as the nature of the sampling means that material from the lids is progressively removed from the experiment. Each treatment was deployed adjacent to one another on a grassy field under ownership of the University of Nottingham in Sutton Bonington, UK (temperate climate) in October 2021 (52°50’05.53“ N, 01°14’53.19” W). No permits were therefore required for field site access. The soil was a gravelly loam; it was freely draining and slightly acidic [17]. Buried samples were buried to 10 cm depth to provide a standardised depth for the samples that would receive minimal UV radiation. The soil temperature of each treatment was monitored using a temperature probe and data logger (Tinytag, Gemini Ltd.), recording measurements hourly and taking a daily average. Site UV-B fluctuations were logged using a UV-B radiation sensor and data logger (SKU 430, Skye Instruments Ltd.), taking measurements every minute which were then averaged and logged every hour. Average maximum/minimum daily air temperatures and rainfall totals each month were also obtained from the nearby Sutton Bonington weather station (52°49’58.77” N, 01°14’59.97” W) [18]. Field-deployed plastics remained undisturbed except for during monthly sampling and maintenance periods, where some exposure of buried samples to sunlight was necessary for a short period of time while the samples were taken which was unavoidable as part of the study being conducted in the natural environment.

Each month, a disc (~5 mm diameter) was randomly removed using a metal hole punch from each of three lids for each treatment (24 discs total). The discs from each of the three lids were used as PS replicates exposed to the same environmental conditions. The discs were gently cleaned following sampling with water to remove any surface dirt and dried at ambient temperatures. This was considered sufficient to limit any interference to the FTIR signal from contamination while not affecting the polymer itself. The samples were then stored together with any previous samples at an ambient temperature in a dark storeroom before FTIR analysis was conducted (this was variable depending on instrument availability, although samples were typically analysed within 2–3 weeks of sampling), then returned to storage following analysis. All samples from the first 12 months of the experiment were analysed (October 2021-October 2022), then samples from all treatments from every three months between 12–24 months of this ongoing experiment (October 2022 plus January, April, July, and October, all 2023).

### 2.2. FTIR data analysis

Samples were analysed using an FTIR spectrometer (Cary 600 Series, Agilent Technologies) with a potassium bromide (KBr) beamsplitter and an Attenuated Total Reflectance (ATR) module with a germanium (Ge) crystal. A total of 64 scans were used for each acquisition at a resolution of 4 cm^-1^, using absorbance across spectrum wavelengths of 4000−950 cm^-1^. Each sample was scanned in this way a total of six times at randomised points across the disc to generate sample technical replicates. Analysis also included a disc from three PS lids which had not been exposed to the environment (known as month 0), in order to act as a treatment control for comparison to environmentally exposed samples. FTIR spectra were compared using Specsplorer v 0.9.6.6 [19]. In preprocessing, the CO_2_ signal was removed and extended multiplicative signal correction (EMSC) was applied for baseline correction of the spectra. These raw baselined spectra were used for all analyses, and outliers were removed via an isolation forest within Specsplorer prior to any analyses. Of the 1167 spectra collected, 40 were removed from the study as outliers, leaving each treatment with between 11 and 18 FTIR spectra across 3 lid replicates for each month analysed.

Spectra were examined using a principal component analysis (PCA) within Specsplorer, and specific absorbances at the wavenumbers discussed were obtained from the raw baselined datasets. Statistical one-way analysis of variance (ANOVA) and post-hoc Tukey tests were carried out in RStudio using the raw baselined spectra obtained from Specsplorer, after checking for normality by plotting histograms of the residuals. Values used to produce the carbonyl index for each sample were also obtained in this way, and averaged to produce a single value for each treatment each month. By comparing the FTIR absorbance of the carbonyl peak (C = O) to a reference peak (for PS this is often the symmetric CH_2_ stretching peak), the degree of photooxidative degradation can be calculated [20]. The PS carbonyl index (CI) wavenumber values used by Mylläri *et al.* [20] (eq. 1) to examine the photodegradation of PS were adjusted slightly (eq. 2) to account for peak shifts and the baseline correction before application to this dataset as follows:


$$𝐂𝐈=\frac{Abs(1726\;{cm}^{-1})}{Abs(2851\;{cm}^{-1})}$$ (1)

$$𝐂𝐈=\frac{Abs(1718.3\;{cm}^{-1})}{Abs(2850.3\;{cm}^{-1})}$$ (2)

The adjusted carbonyl index was applied to the absorbances of each sample for each treatment to track photooxidative degradation over the full 24-month exposure period. Other studies that seek to build upon the results of the presented here may wish to consider the incorporation of additional analysis such as Rock-Eval pyrolysis, x-ray analysis, and gel permeation chromatography to further investigate plastic degradation processes.

## 3. Results

### 3.1. Polystyrene lid chemistry and natural heterogeneity

**Fig 1:**
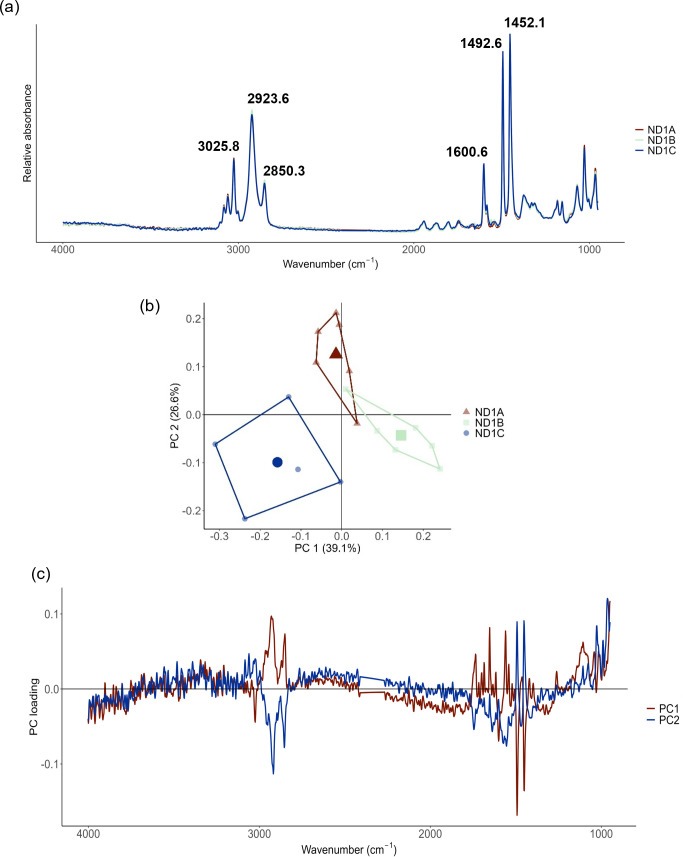
Variation in month 0 (October 2021) FTIR spectra. (a) shows the baselined month 0 polystyrene spectra for each lid sample with key wavenumbers labelled, **(b)** Principal Component Analysis (PCA) of the spectra of the three lid repetitions, explaining 39.1% of variation between samples within PC1, and (c) the principal component loading for the PCA, showing mostly scattered variation in lid chemistry across the absorbance spectrum. Greatest influences on the PCA were within aromatic peak wavenumber regions and was greatest at wavenumber 1492.6 cm^-1^.

Samples were taken from three virgin cup lids that were not exposed to the experimental environment (known as month 0) and analysed using FTIR to generate a treatment control and assess any natural chemical heterogeneity between samples owing to production processes. All three spectra contained the expected main absorbance peaks for PS at wavenumbers 2923.6 cm^-1^ and 2850.3 cm^-1^ in relation to C-H stretch, wavenumber 3025.8 cm^-1^ in relation to C-H of the aromatic ring, and at wavenumbers 1600.6 cm^-1^, 1492.6 cm^-1^, and 1452.1 cm^-1^ in relation to C = C of the aromatic ring (Fig 1a) [21,22]. Absorbance peaks in the wavenumber region 1400−950 cm^-1^ also confirmed that the PS structure was amorphous [23].

A PCA to compare month 0 lid FTIR spectra within and between lid samples was able to explain 39.1% of variation across principal component one (PC1) and 26.6% across principal component two (PC2) (Fig 1b). There was some clustering of spectra for each lid across the PCA, indicating a small amount of natural chemical variation in the composition between each lid. Investigation of the component loading for PC1 indicated mostly scattered variation across the absorbance spectrum, which can be attributed to noise and natural fluctuations in moisture levels within the lab that are then detectable within the FTIR spectra (Fig 1c). Some of the larger influences on PC1 loadings within the aromatic wavenumber regions could, however, be related to some natural heterogeneity. Influence was greatest at wavenumber 1492.6 cm^-1^, so an ANOVA to compare the raw absorbance at this wavenumber for each spectrum was performed. The ANOVA indicated that there was a significant difference in absorbance between spectra at this wavenumber (p = 0.005, F = 7.83), suggesting natural chemical variability in the PS lids owing to production processes. As a result, samples from three separate lids were analysed each month to account for differences in absorbance relating to natural chemical variability of the lids.

### 3.2. Climate data

**Fig 2:**
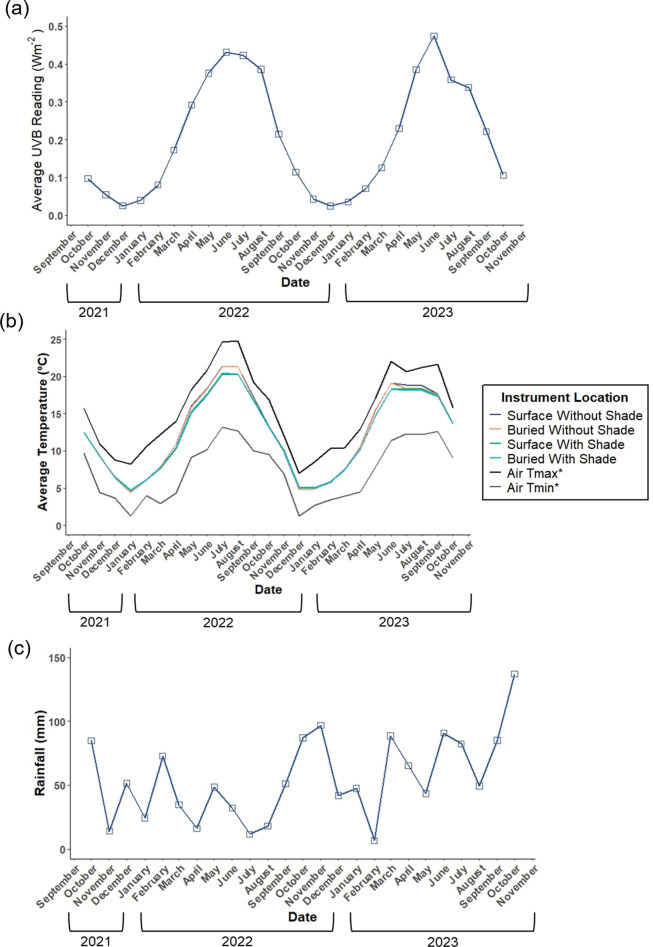
Site climatic variation through time over the 24-month sampling period October 2021-October 2023. (a) shows average UVB fluctuations at the site, (b) average soil temperature recorded at each treatment, alongside average air Tmax and Tmin recorded at the nearby Sutton Bonington weather station, (c) the average rainfall recorded at the nearby Sutton Bonington weather station. Data marked with * in (b) and all data in (c) were obtained from the Met Office (2022).

Monthly changes in both UV-B radiation and temperature reflected typical seasonal weather cycles, with both variables declining over autumn and winter months and increasing across spring and summer (Fig 2a and 2b). Differences in soil temperature between treatments were greatest in the summer period, with shaded treatments recording lower average soil temperatures than those not under shade. Monthly total rainfall fluctuated throughout the 24-month period with no clear trend or cycle (Fig 2c).

### 3.3. Degradation between treatments

**Fig 3:**
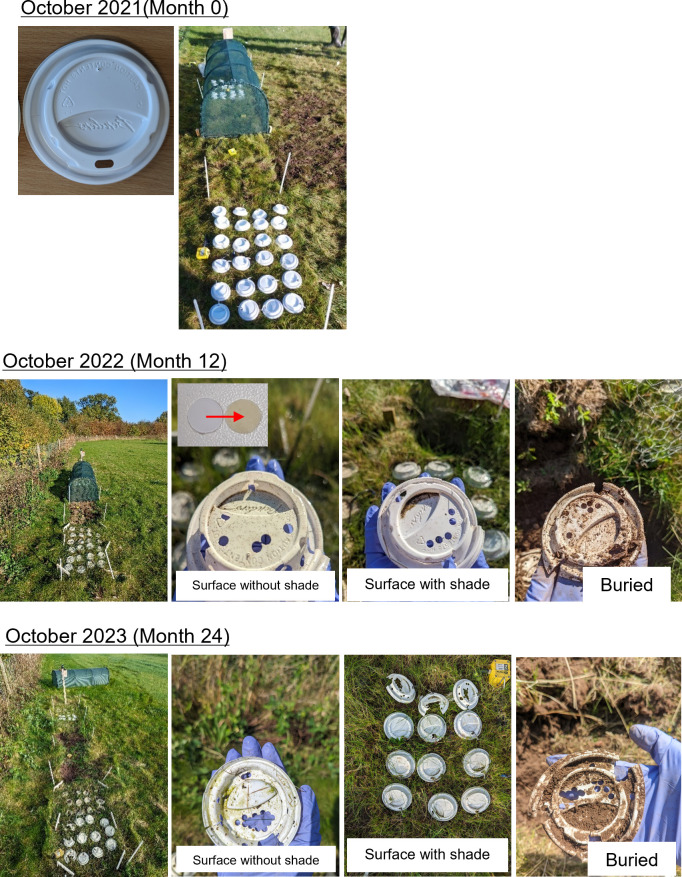
Images of the cup lids during each year of the experiment. The inset image on the top surface without shade sample picture shows a visual comparison between a sample taken during the sampling period from that treatment (right) to an unexposed (month 0) PS cup lid sample.

All PS samples remained present and visually recognisable after 24 months of environmental exposure (Fig 3). Buried samples appeared largely unchanged after the duration of the experiment aside from a flattening of their 3D form. In contrast, while surface samples retained their 3D morphology, the lids in these treatments had experienced some degree of yellowing and were found to be more brittle (at least qualitatively). A significant biofilm was also visually noticeable on the underside of surface samples towards the end of the second summer (2023).

**Fig 4:**
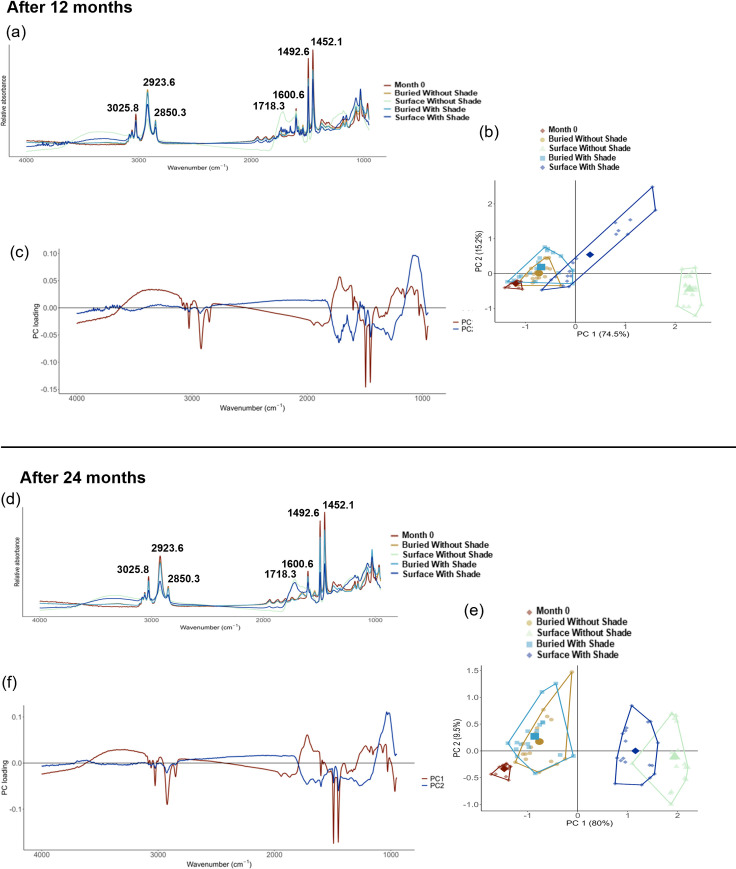
Variation in FTIR spectra between treatments after (a-c) the first 12 months, and (d-f) 24 months, of environmental exposure. (a, d) depict baselined PS spectra showing how month 0 samples compared to samples from each treatment after each exposure period with key wavenumbers labelled, (b, e) contain PCAs of the treatment scans, and (c, f) show the principal component loading for the respective PCAs in (b) and **(e)**.

Following 24 months of environmental exposure, each treatment showed some variation in absorbance at peaks across their FTIR spectra, albeit to differing degrees (Fig 4). Surface without shade spectra appeared to change the most, showing both changed absorbance at the native wavenumber peaks and also the presence of a new peak at wavenumber 1718.3 cm^-1^, the wavenumber associated with the carbonyl peak (C = O) resulting from photooxidation. This carbonyl peak was also present for surface with shade spectra after 24 months of exposure, but the peak had not formed after the first 12 months (Fig 4a and 4d). Similarly, there were some signs of distortion in the spectra that appeared after 12 months for surface without shade spectra and after 24 months for surface with shade spectra, which could relate to changes in the surface colouration or chemical composition that affected the reflectance of the IR beam. This distortion was visible in the presence of a shallow broad peak between wavenumbers 3662.2 cm^-1^ and 3122.2 cm^-1^, and also some distortion between 2462.7 cm^-1^ and 1835.0 cm^-1^. Although outside the scope of this experiment, this effect should be investigated further as it could have influence on the degradation rate of PS.

To explore the degradation trends further, the spectra for each treatment was explored and compared to other treatments and month 0 using a PCA following both 12 and 24 months of environmental exposure. After 12 months of exposure, the PCA PC1 explained 74.5% of the variation in the spectra, while PC2 explained 15.2% (Fig 4b). Across PC1, both buried treatment spectra were overlapping and most similar to month 0 spectra, with month zero spectra clustered to the left of PC1 and the buried spectra clustering close to this. Surface without shade spectra were the most different to month 0, clustering to the far right of PC1, with shaded with shade spectra showing a spread in variation across the middle of the PCA. The majority of the spectra clustering to the right of the surface with shade treatment were from the same lid sample, indicating a difference in the spectra within the same treatment and thus the degradation of the lids. As it explained most of the variation between the spectra, the component loading of PC1 was examined further, showing that the most variation informing PC1 was at wavenumbers 1492.6 cm^-1^ and 1452.1 cm^-1^ which were two of the main wavenumbers corresponding to the C = C bond of the aromatic ring (Fig 4c). The raw baselined spectra at these two wavenumbers were then compared in ANOVAs, revealing a significant difference between treatments at both wavenumbers (p < 0.001, group degrees of freedom = 4 and residual degrees of freedom = 83 for both tests, F = 106.10 and R^2^ = 0.84 at 1492.6 cm^-1^ and F = 81.28 and R^2^ = 0.80 at 1452.1 cm^-1^). Post-hoc Tukey tests showed significant differences between some treatments and not others (Table 1). In particular, it revealed that all treatments were significantly different to month zero samples at these wavenumbers, and absorbances for surface without shade samples after 12 months exposure were also significantly different from all other treatments.

**Table 1:** Post-hoc Tukey test outputs of an ANOVA to compare FTIR absorbance between treatments and month 0 (October 2021) at the two wavenumbers (1492.6 and 1452.1) that showed most variation after 12 months environmental exposure.

	Month 0		Buried Without Shade		Surface Without Shade		Buried With Shade		Surface With Shade	
	1492.6	1452.1	1492.6	1452.1	1492.6	1452.1	1492.6	1452.1	1492.6	1452.1
**Month 0**	N/A	N/A	<0.001	<0.001	<0.001	<0.001	<0.001	<0.001	<0.001	<0.001
**Buried Without Shade**	<0.001	<0.001	N/A	N/A	<0.001	<0.001	0.997	0.9966	0.120	0.0363
**Surface Without Shade**	<0.001	<0.001	<0.001	<0.001	N/A	N/A	<0.001	<0.001	<0.001	<0.001
**Buried With Shade**	<0.001	<0.001	0.997	0.9966	<0.001	<0.001	N/A	N/A	0.257	0.0968
**Buried With Shade**	<0.001	<0.001	0.120	0.0363	<0.001	<0.001	0.257	0.0968	N/A	N/A

**Fig 5:**
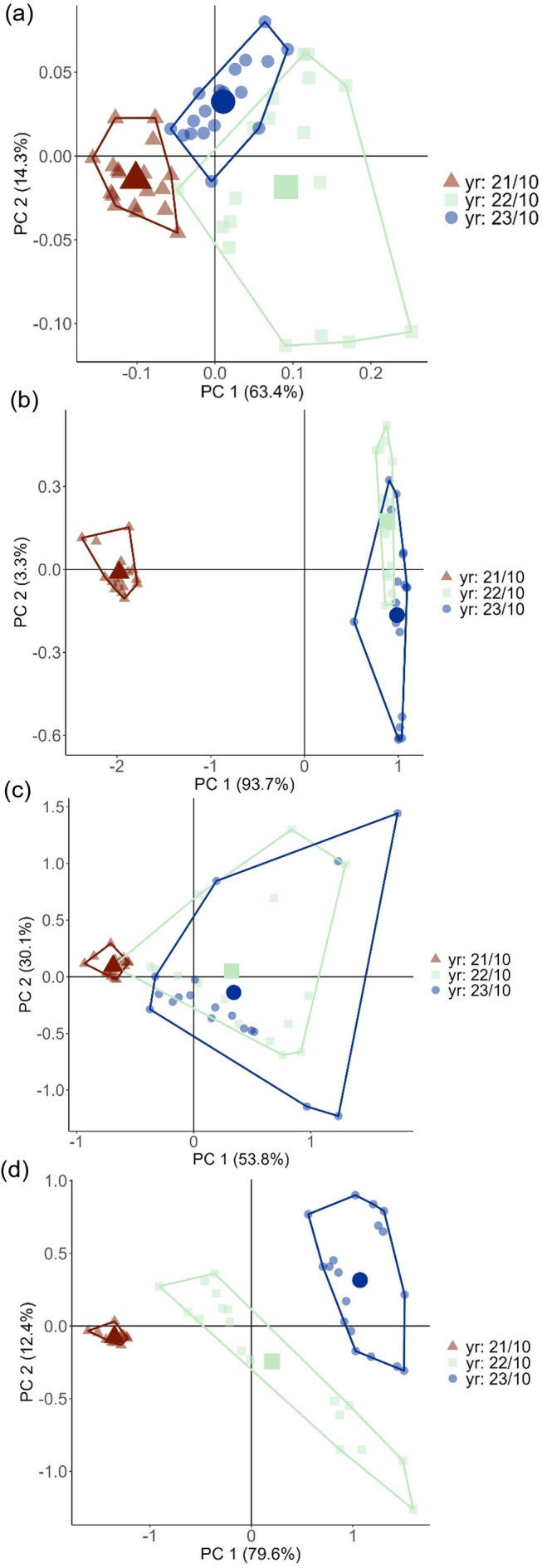
2D Principal component analyses showing yearly FTIR spectra variation in treatments across the whole 24-month sampling period from October 2021 (month 0)-October 2023. (a) buried without shade treatment spectra, (b) surface without shade FTIR spectra, (c) buried with shade treatment spectra, and (d) surface with shade spectra.

After 24 months of environmental exposure, a PCA explaining 80% of variance across PC1 and 9.5% across PC2 was generated (Fig 4e). Again, both buried treatment spectra were overlapping and the most similar to month 0 spectra, although there was slight clustering away from month 0, suggesting some variation in the chemistry. In contrast, both surface treatments were clustered to the right of PC1, with some overlapping, indicating that the two treatments had chemistries most different from month 0. As with the PCA after 12 months of exposure, the component loading of PC1 indicated the most variation at the C = C aromatic bond (wavenumbers 1492.6 cm^-1^ and 1452.1 cm^-1^) (Fig 4f). Statistical ANOVA tests to compare absorbance between treatments at these wavenumbers indicated a significant difference between treatments at both wavenumbers (p < 0.001, group degrees of freedom = 4 and residual degrees of freedom = 84 for both tests, F = 414.49 and R^2^ = 0.95 at 1492.6 cm^-1^ and F = 309.37 and R^2^ = 0.94 at 1452.1 cm^-1^). Post-hoc Tukey tests showed significant differences in absorbance at both wavenumbers lay between all treatments except buried without shade and buried with shade which were not significantly different to one another (Table 2). Summary annual PCA plots for each treatment can be found in Fig 5.

**Table 2:** Post-hoc Tukey test outputs of an ANOVA to compare FTIR absorbance between treatments and month 0 (October 2021) at the two wavenumbers (1492.6 and 1452.1) that showed most variation after 24 months environmental exposure.

	Month 0		Buried Without Shade		Surface Without Shade		Buried With Shade		Surface With Shade	
	1492.6	1452.1	1492.6	1452.1	1492.6	1452.1	1492.6	1452.1	1492.6	1452.1
**Month 0**	N/A	N/A	<0.001	<0.001	<0.001	<0.001	<0.001	<0.001	<0.001	<0.001
**Buried Without Shade**	<0.001	<0.001	N/A	N/A	<0.001	<0.001	0.663	0.833	<0.001	<0.001
**Surface Without Shade**	<0.001	<0.001	<0.001	<0.001	N/A	N/A	<0.001	<0.001	<0.001	<0.001
**Buried With Shade**	<0.001	<0.001	0.663	0.833	<0.001	<0.001	N/A	N/A	<0.001	<0.001
**Buried With Shade**	<0.001	<0.001	<0.001	<0.001	<0.001	<0.001	<0.001	<0.001	N/A	N/A

### 3.4. Changes in degradation rate over time

**Fig 6:**
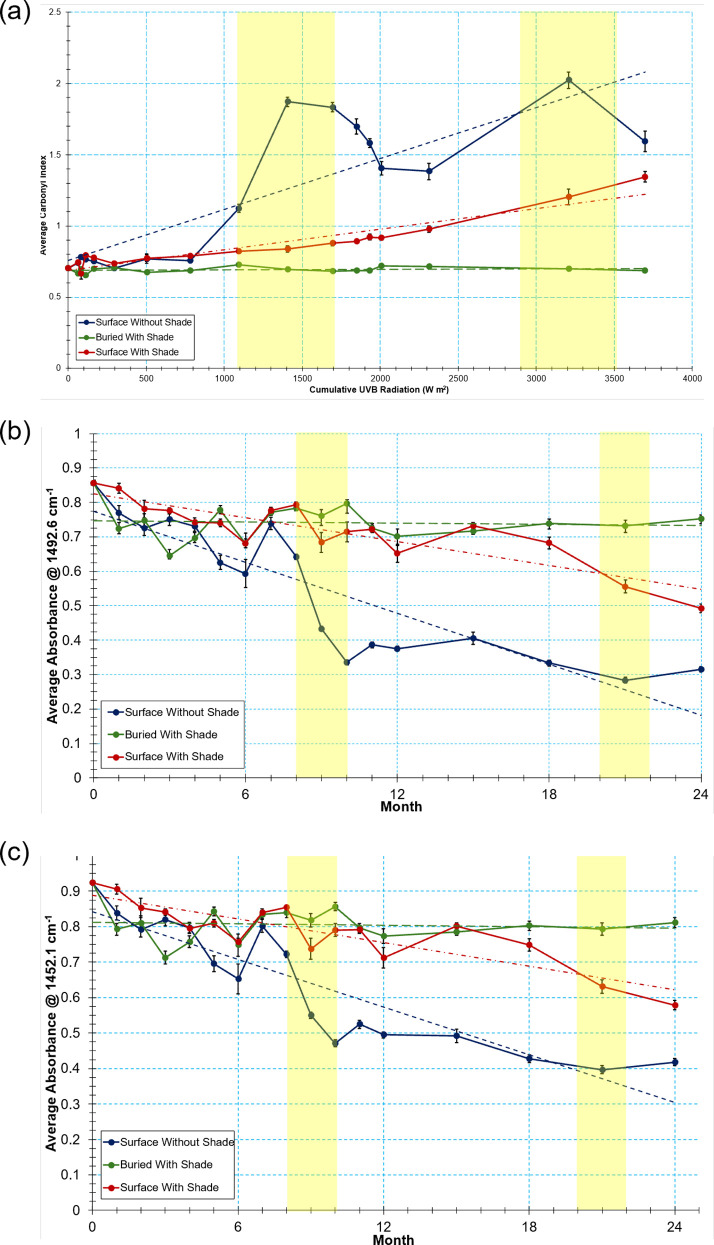
FTIR spectral absorbance through time at (a) wavenumber 1492.6 cm^-1^ and (b) 1452.1 cm^-1^ (c) Average FTIR carbonyl index with cumulative UVB radiation across the 24-month sampling period. Cumulative UVB radiation over the sampling period was calculated by summing the average UVB radiation every hour for each month of the experiment. For all, error bars depict the standard error of the mean, and bands showing absorbance within the summer months are highlighted. Note that FTIR spectra were obtained for each month of the first 12 months of the experiment, then every three months thereafter.

The degradation rate of each treatment over the course of the experiment was examined further through a series of scatterplots and ANOVA regressions to track average absorbance through time at the two most variable wavenumbers identified by the PCAs in Fig 4 (wavenumbers 1492.6 cm^-1^ and 1452.1 cm^-1^ relating to the C = C aromatic bond) (Fig 6a, 6b). Since there was significant overlap in the PCAs between both of the buried treatment spectra, only the degradation of buried with shade treatment was investigated further under the assumption that the two treatments behaved in a similar way. Trend direction for all three treatments were very comparable between the two wavenumbers, but variation within the trends differed between treatments. For surface without shade treatment spectra, the average absorbance began to decline through the first spring (2022), and there was a clear drop in the absorbance in the first summer (May-August 2022 or months 7–10) coinciding with the seasonal rise of UV-B and temperature, indicating possible seasonal influence on degradation rate. There was also a decrease in absorbance at both wavenumbers during the second summer, although this was less pronounced. An ANOVA regression across the entire 24-month period indicated a significant difference between months at both wavenumbers (p < 0.001, group degrees of freedom = 16 and residual degrees of freedom = 268 for both tests, F = 135.84 and R^2^ = 0.89 at 1492.6 cm^-1^ and F = 95.20 and R^2^ = 0.85 at 1452.1 cm^-1^) For surface with shade spectra, there was a more gradual decline in the absorbance through time at both wavenumbers, although there was a slightly more pronounced decrease in absorbance during the second summer (2023). An ANOVA regression also indicated a significant difference between months at both wavenumbers (p < 0.001, group degrees of freedom = 16 and residual degrees of freedom = 279 for both tests, F = 30.69 and R^2^ = 0.64 at 1492.6 cm^-1^, and F = 30.22 and R^2^ = 0.63 at 1452.1 cm^-1^). For buried with shade samples, there was no clear change in the absorbance at either wavenumber through time across the experiment. The ANOVA did identify a significant difference between months for both wavenumbers (p < 0.001, group degrees of freedom = 16 and residual degrees of freedom = 280 for both tests, F = 9.14 at 1492.6 cm^-1^ and F = 8.35 at 1452.1 cm^-1^), but the lower R^2^ suggested limited variation in absorbance could be consistently explained by differences between months in comparison to the other treatments (R^2^ = 0.34 at 1492.6 cm^-1^ and R^2^ = 0.32 at 1452.1 cm^-1^).

### 3.5. Changes in carbonyl index

Average carbonyl index values for each month of each treatment were plotted against cumulative UV-B to show photooxidative degradation trends over the 24-month sampling period (Fig 6c). Cumulative UV-B was calculated by summing the average UV-B radiation every hour for each month of the experiment. The carbonyl index did not significantly fluctuate for the buried with shade PS treatment spectra over time, with a maximum range of 0.07, indicating little photooxidative degradation. An ANOVA regression did identify a significant difference in carbonyl index between months (p < 0.001, group degrees of freedom = 16 and residual degrees of freedom = 280, F = 4.19), but a low R^2^ suggested limited variation in absorbance could be consistently explained by differences between months for this treatment (R^2^ = 0.19). Trends were more apparent for the surface PS lid treatments. For these, the carbonyl index initially fluctuated similarly to buried samples for the first winter months October 2021-March 2022 (months 1–5), corresponding with low UV radiation levels during this period. From April 2022 (month 6) where cumulative UV radiation reached 503.58 W m^-2^, the carbonyl index values for the two surface treatments began to noticeably deviate from the buried treatment. For surface without shade sample spectra, there was a significant increase in the carbonyl index between May and July 2022 (months 7–9) which corresponded to the steep rise of UV-B radiation (ranging from a cumulative value of 783.38 W m^-2^ in May to 1407.05 W m^-2^ by July) during summer months, suggesting these samples experienced the most photooxidative degradation (in agreement with the decreased absorbance at native peaks for this treatment). This then decreased until January 2023 (month 15) which coincided with the period of falling and low UV-B radiation and temperature approaching and during winter months. A similar trend to the first summer then followed as carbonyl index rose with a spike in July 2023 (month 21) before decreasing to the last sampling date October 2023 (month 24), as was the case during the first 12 months of the exposure period. Overall, surface without shade samples had a maximum carbonyl index range of 1.32. An ANOVA regression identified a significant difference in carbonyl index between months for this treatment (p < 0.001, group degrees of freedom = 16 and residual degrees of freedom = 268, F = 166.19) with a high R^2^ of 0.91. For surface with shade samples, there was a more gradual increase in the carbonyl index through time which was slightly greater during the second year of the experiment, indicating a more gradual photooxidative degradation of these coffee cup lid treatment samples. There was a maximum range of 0.68 in the carbonyl index for surface with shade samples over the 24-month exposure, and an ANOVA regression also identified a significant difference in carbonyl index between months for this treatment (p < 0.001, group degrees of freedom = 16 and residual degrees of freedom = 279, F = 58.66) but with a slightly lower R^2^ of 0.77.

As the samples with most variation through the experiment, the average carbonyl index values of surface without shade samples were investigated further via a multiple regression ANOVA analysis to identify any correlations with average site UVB radiation, soil temperature, and rainfall rate across the first twelve months of the experiment (month 0 to month 12, the period where regular monthly measurements were taken). The regression identified an almost significant coefficient between carbonyl index and UVB radiation (p = 0.079, F = 3.75, degrees of freedom = 12). This model was improved by the addition of soil temperature (p = 0.058, F = 3.83, degrees of freedom = 12), and subsequently worsened by the inclusion of rainfall rate (p = 0.134, F = 2.42, degrees of freedom = 12). Soil temperature alone, however, was found to be the most significant predictor (p = 0.002, F = 17.16, degrees of freedom = 12), and this may suggest that soil temperature is a more stable proxy measurement of seasonal variation compared to a more fluctuating UVB radiation rate over time.

## 4. Discussion

This study aimed to elucidate the effect of natural UV exposure in a temperate climate on the photooxidative degradation rate of PS. Following 24 months of environmental exposure, all PS lids in all treatments were still present, and all samples showed limited signs of significant visible degradation compared to month 0, aside from the yellowing and embrittlement of surface treatment samples. Chemically, each treatment showed variable signs of degradation, with statistically significant differences in absorbance at the most variable native PS peaks identified between the treatments compared to month 0 after both 12 and 24 months. Surface treatment samples, particularly surface without shade samples showed the most variation from month 0 samples in PCAs, while buried treatment samples remained most similar to month 0 samples after both 12 and 24 months. Separate comparisons of each treatment across each sampling month showed a statistically significant difference in absorbance at native wavenumber peaks and at the carbonyl index for all treatments, suggesting some degradation of all samples. Higher R^2^ values for surface treatments indicated the most variation in absorbance could be explained by differences between the months for these treatment types and this was mirrored in the PCAs. Exposure to UV has previously been shown to cause photooxidative degradation of PS in accelerated laboratory studies [12,24]. Particularly in aromatic regions as these contain delocalised electrons that require less UV energy to enter an excited state compared to other bonds [9,10,25]. The present study indicated that most variation in absorbance was located at wavenumbers associated with aromatic regions (namely wavenumbers 1492.6 cm^-1^ and 1452.1 cm^-1^), and that samples exposed to natural irradiance experienced the most photooxidative degradation. This is therefore in agreement with the hypothesis that PS samples exposed to natural irradiance would experience more degradation via photooxidative mechanisms than those buried in soil.

Examination of changes in absorption at the two most variable native aromatic wavenumbers and the carbonyl index through time suggested that degradation was initiated within as little as one month of the experiment for surface samples, although significant changes in the chemical composition began to occur in late spring and summer of the first experiment year. Signs of continued degradation of the spectra were limited in winter months, but the accelerated summer degradation trend repeated during the second summer, indicating a seasonal influence on the degradation rate whereby the degradation is not constant through time. Rainfall fluctuated significantly over time and was found via the multiple regression to be unlikely to have had influence on the degradation rate via influences on the carbonyl index. However, both UV-B and temperature exhibited similar seasonal trends to the degradation rate of surface treatments, especially to the surface without shade spectra, whereby increases in temperature and UVB during summer months coincided with increases in PS degradation. The inclusion of UVB and temperature in the multiple regression as predictors of carbonyl index (and therefore photooxidative degradation rate) for the surface without shade treatment indicated a possible influence of these factors on the degradation rate, providing support for the hypothesis that seasonal variation in irradiation leads to variation in the degradation rate of PS.

While ambient temperature alone was the most significant predictor, it was unlikely to have been able to supply sufficient energy to initiate thermal degradation within this experiment (PS thermal degradation begins at 270°C in air) [26]. However, exposure to UV radiation has been found to trigger temperature-related degradation as long as oxygen is available. This suggests that the two factors in combination are likely to contribute to degradation through photooxidative degradation. This is in agreement with previous studies comparing the degradation rates of plastics exposed to air and seawater treatments, where seawater samples were exposed to less sunlight and oxygen, and therefore degraded the least [14,16,27].

An additional seasonal trend was apparent for surface without shade treatment samples that was not directly linked to an increase in UV radiation and temperature during summer months. At the end of each summer period, the carbonyl index decreased for surface without shade treatment lid spectra; this seasonal trend was not mirrored by an increase in absorption at the aromatic ring wavenumber peaks. This seems to indicate an overall decrease in the number of carbonyl groups in the polymer and therefore the loss of some of these carbonyls from the polymer itself. Although biodegradation is thought to operate at speeds several orders of magnitude slower than photooxidation, the possible role of biotic processes (as well as other unknown processes not considered here) in the degradation of PS samples cannot be completely ruled out as such processes could give some explanation for the related decrease in carbonyl compounds and the degradation of buried samples [15,28].

For example, once abiotic oxidation products are formed, microorganisms within a biofilm can then (amongst other processes) consume these to ultimately form carbon dioxide and water as end products, resulting in a drop in the number of carbonyl groups in the polymer, known as biodegradation [8,25,28]. It may be that during these periods the consumption of carbonyl groups by microorganisms could be greater than their production by photo-oxidative processes, resulting in an overall decrease in the number of carbonyl groups in the polymer. However, there is variable existing evidence with regards to whether PS experiences significant levels of biodegradation which would be noticeable over the timeframe used in this study [29–32]. This relationship should therefore be explored further as changes in the composition of PS and its bioavailability is likely to alter the way it both reacts to and interacts with the environment throughout its lifetime. It also remains unclear whether such chemical changes resulting from photooxidative degradation affect the environmental toxicity of PS, although the decreased chemical structural integrity is likely to have at least led to the visible changes in mechanical integrity of the lids.

## 5. Conclusions

This study has demonstrated that UV photooxidative mechanisms were the most significant contributors to the chemical degradation of PS coffee cup lids over 24 months of exposure to a temperate environment. Exposure to UV irradiance is therefore important for the initiation of PS degradation released into the environment, and this will be important when considering the environmental fate of PS waste. Environmental degradation was also not continuous and varied seasonally according to the degree of UV and temperature exposure, giving credence to the notion that the specific degradation rate of PS waste will vary depending on the specific conditions that the plastic is exposed to during its lifetime. There was also some indication that the environmental degradation of PS was the result of a complex interaction between abiotic and biotic factors which were ultimately controlled by the seasonal exposure of the PS to UV irradiance. As the experiment continues to progress, these interactions may be of increasing relevance to the ongoing degradation of these samples and should be investigated further.

## Supporting information

S1 File FTIR Spectral Data. Data collected and analysed in the study.
(CSV)

S2 File FTIR Metadata. Metadata used to support the data used in S1.
(CSV)
